# Validity and reliability of the Dutch translation of the OPUS’ client satisfaction with device module in chronic users of hand orthoses

**DOI:** 10.1186/s12955-023-02181-3

**Published:** 2023-08-21

**Authors:** Tanja Oud, Jana Tuijtelaars, Jimmy Schenk, Frans Nollet, Merel-Anne Brehm

**Affiliations:** 1 Amsterdam UMC, Department of Rehabilitation Medicine, University of Amsterdam, Meibergdreef 9, Amsterdam, The Netherlands; 2 Amsterdam Movement Sciences, Rehabilitation & Development, Amsterdam, The Netherlands; 3 Amsterdam UMC, Department of Epidemiology and Data Science, Amsterdam Public Health, University of Amsterdam, Meibergdreef 9, Amsterdam, The Netherlands; 4 Department of Anesthesiology, Amsterdam Cardiovascular Sciences, Amsterdam UMC, University of Amsterdam, Meibergdreef 9, Amsterdam, The Netherlands; 5 Department of Intensive Care, Amsterdam Cardiovascular Sciences, Amsterdam UMC, University of Amsterdam, Meibergdreef 9, Amsterdam, The Netherlands

**Keywords:** Patient reported outcome measures, OPUS-CSD, Reliability and validity, Orthotic Devices, Hand

## Abstract

**Background:**

Orthosis satisfaction is an important outcome in assessing quality of care. However, no measurement specifically assessing orthosis satisfaction is available in the Dutch language. Therefore, the aim of this study was to translate the Client Satisfaction with Device (CSD) module of the Orthotics and Prosthetics Users’ Survey (OPUS) into Dutch, and to assess its content validity, structural validity and reliability in persons with chronic hand conditions.

**Methods:**

The CSD was translated and cross-cultural adapted according to respective guidelines. To determine content validity, 10 chronic hand orthotic users and two professionals judged the relevance, comprehensibility, and comprehensiveness of the Dutch CSD (D-CSD). Thereafter, in a cross-sectional study, 76 persons were asked to complete the D-CSD twice, with a 2-week interval. Dimensionality of the D-CSD was examined by principal component analysis (PCA), and factor model fit was assessed by confirmatory factor analysis (CFA). Reliability was assessed as internal consistency and test-retest reliability, including the 95% limits of agreement (LoA), the standard error of measurement (SEM) and smallest detectable change (SDC).

**Results:**

The D-CSD items and response options were deemed relevant and comprehensible. After adding an item on cleaning the orthosis, content validity was judged sufficient. PCA indicated a one-factor model, which was confirmed by CFA. We found good internal consistency (Cronbach’s alpha = 0.82; 95%CI 0.75–0.87), and moderate to good test-retest reliability (ICC = 0.81; 95%CI 0.71–0.87). There was no difference between the mean D-CSD score at test (26.8 points) and retest (25.9 points) (mean (SD) difference: 0.86 points (4.00); 95%CI -0.06-1.79; p = 0.07). The 95% LoA were −6.99 to 8.71, and the SEM and SDC were 2.88 and 7.98 points, respectively.

**Conclusions:**

Based on sufficient content and structural validity, and good reliability, we consider the D-CSD a useful tool to evaluate orthosis satisfaction in persons with chronic hand conditions on group level. Because of a relatively high SDC, sensitivity to detect changes over time on individual level is limited.

**Study registration number:**

NCT05320211.

## Introduction

Chronic hand conditions often lead to life-long impairments, such as pain, muscle weakness, spasticity, and joint and/or muscle contractures [1–3]. These impairments limit a person’s ability to perform activities of daily living [3–5], and negatively impact quality of life [4, 6]. Hand orthoses are applied to reduce hand impairments, and to improve performing daily activities [7–10]. Annually, about 27,400 upper extremity orthoses are provided in the Netherlands [11], of which a large proportion comprises hand orthoses.

Most people with chronic hand conditions usually wear a custom-fabricated orthosis on a daily basis, which emphasizes the importance to assess a person’s experiences with the quality of care. A relevant patient-reported outcome regarding the quality of care is orthosis satisfaction, which, over the past years, has increasingly received attention in the field of orthotics. However, only two of the questionnaires evaluating orthosis satisfaction showed adequate construct validity for upper extremity orthoses [12], namely the Client Satisfaction with Device (CSD) module [13, 14] and the Quebec User Evaluation of Satisfaction with assistive Technology (QUEST) 2.0 [15, 16].

The CSD is one of the five modules of the Orthotics and Prosthetics Users’ Survey (OPUS), assessing persons’ satisfaction with their orthotic or prosthetic device [17]. The CSD has shown good internal consistency and test-retest reliability in spine, and upper and lower extremity orthotic and prosthetic users [14, 18–23], and moderate test-retest reliability in lower extremity prosthetic users [24]. The QUEST 2.0 evaluates satisfaction within a wide range of assistive devices and consists of a device and services subscale. The device subscale has shown good internal consistency and test-retest reliability [25]. The QUEST 2.0 has been translated and validated in Dutch (D-QUEST) [15].

The CSD and (D-)QUEST 2.0 have only three aspects in common: weight, comfort and durability. Furthermore, the QUEST was developed as a generic tool for a variety of assistive devices, while the CSD was specifically developed for orthotic and prosthetic devices, addressing for example fitting, skin reactions and aesthetics. Therefore, the CSD and QUEST complement each other when evaluating satisfaction with hand orthoses.

The CSD has originally been developed in English [13, 17], and was translated and validated into various different languages [14, 18–23, 26, 27], however so far not in Dutch. Therefore, the aim of this study was to (1) translate and cross-culturally adapt the CSD into the Dutch language, and (2) assess the content validity, structural validity and reliability of the Dutch CSD (D-CSD) in chronic hand orthotic users. Considering the good psychometric properties of the other CSD translations [14, 18–23], we hypothesized that the D-CSD would be a valid and reliable tool to measure orthosis satisfaction in our population.

## Methods

### Study design

This cross-sectional study was conducted according to the COnsensus-based Standards for the selection of health Measurement Instruments (COSMIN) Study Design checklist and reported following the COSMIN reporting guideline [28, 29]. First, we translated and cross-culturally adapted the CSD into the Dutch language, and subsequently, we evaluated the content validity of the D-CSD. Second, we assessed the structural validity and reliability of the D-CSD in chronic hand orthotic users.

### Translation and assessment of content validity

#### Questionnaire

The CSD is a self-administered questionnaire that originally consists of 11 items [17]. Since two items (both dealing with the costs of the devices) did not fit the construct, and these items are irrelevant to the Dutch population because orthotics are reimbursed by the health insurance, we used the 9-item version of the CSD as proposed by Jarl et al [26]. Items were rated on a 5-point Likert scale, ranging from 0=‘strongly disagree’ to 4=‘strongly agree’. The response option ‘Don’t know/Not applicable’ was not scored numerically. The sum score was calculated using the following formula:

$$(\sum \text{i}\text{t}\text{e}\text{m}\ \text{s}\text{c}\text{o}\text{r}\text{e}\text{s})\times \frac{\text{t}\text{o}\text{t}\text{a}\text{l}\ \text{n}\text{o}. \text{o}\text{f}\ \text{i}\text{t}\text{e}\text{m}\text{s}}{\text{n}\text{o}. \text{o}\text{f}\ \text{i}\text{t}\text{e}\text{m}\text{s}\ \text{s}\text{c}\text{o}\text{r}\text{e}\text{d}\ \text{n}\text{u}\text{m}\text{e}\text{r}\text{i}\text{c}\text{a}\text{l}\text{l}\text{y}}$$

#### Translation procedure

The CSD was translated in accordance with the guidelines for translation and cross-cultural adaptation [30]. Two native Dutch speakers (TO and RdJ) independently translated the CSD from English into Dutch. TO was aware of the examined concepts, while RdJ was not aware of the concepts, and had no medical background. Both translators and an independent person (MB) discussed differences between the two translations to reach consensus. Two native English speakers without medical background (AN and JN) independently translated the consensus version from Dutch into English. To avoid information bias, both translators were unaware of the concepts explored. Taking all translations into consideration, an expert committee (i.e. all translators, a methodologist and health care professionals) consensually agreed on a pre-final version of the D-CSD. The developer of the CSD was asked for feedback, and relevant adjustments were made to finalize the D-CSD. Finally, the D-CSD was pretested by determining the content validity in chronic hand orthotic users. Content validity is ‘the degree to which the content of an instrument is an adequate reflection of the construct to be measured’ [31].

#### Study population

According to the COSMIN guidelines, at least 7 participants are needed to determine content validity [28]. We included 10 chronic hand orthotic users, who were recruited from our outpatient rehabilitation clinic database at Amsterdam UMC, location Academic Medical Center. Eligible participants were characterized as (1) being ≥ 18 years, (2) having a stable, chronic hand condition due to an injury, or a musculoskeletal, neuromuscular or neurological disorder, and (3) permanently wearing a thumb, wrist, or wrist-thumb orthosis for ≥ 3 months, custom-fabricated by an orthopedic company (OIM Orthopedie, the Netherlands). Participants were excluded when they wore (1) an orthosis for a dysfunctional hand, (2) a broken orthosis, (3) a night orthosis, and (4) had insufficient mastery of the Dutch language.

#### Procedure content validity

After signing informed consent, demographics and clinical characteristics of the participants were obtained. Subsequently, the content validity of the D-CSD was evaluated by cognitive debriefing. Content validity was judged based on 10 criteria related to relevance, comprehensiveness and comprehensibility (Table 1) [32]. We did not assess criterion 5 (appropriateness of the recall period) since the D-CSD aims to evaluate orthosis satisfaction at present time. Cognitive debriefing was performed by TO and JT using a Three-Step-Test-Interview [33]. Interviews were video-recorded with Microsoft TEAMS. TO and JT additionally judged the relevance, comprehensiveness and comprehensibility of the D-CSD, except for criteria 7 and 8.

**Table 1:** Ten criteria for rating content validity

**Relevance**
1. Are the included items relevant for the construct of interest? 2. Are the included items relevant for the target population of interest? 3. Are the included items relevant for the context of use of interest? 4. Are the response options appropriate? 5. Is the recall period appropriate?
**Comprehensiveness**
6. Are no key concepts missing?
**Comprehensibility**
7. Are the patient-reported outcome measure (PROM) instructions understood by the population of interest as intended? 8. Are the PROM items and response options understood by the population of interest as intended? 9. Are the PROM items appropriately worded? 10. Do the response options match the question?

### Data analysis

Socio-demographics and clinical characteristics of participants were summarized with descriptive statistics. The interviews were transcribed verbatim using MAXQDA 2022. For each participant, information on each criterion was highlighted and recoded into a positive (i.e. agreed with criterion) or negative (i.e. disagreed with criterion) score by two researchers. Each criterion was than rated sufficient (≥ 85% of the participants agreed with the criterion) or insufficient (< 85% of the participants agreed with the criterion) [32, 34].

If the analysis of comprehensiveness pointed out that a certain aspect of orthosis satisfaction was missed, as reported by ≥ 25% of the participants, we formulated an additional item. Thereafter, the comprehensibility and relevance of this item were assessed in three other participants. Subsequently, a rating for relevance, comprehensiveness, and comprehensibility was determined by summarizing the criteria ratings given by the participants and professionals for each component. Content validity was judged sufficient if all three components were rated positive [32, 34].

### Assessment of structural validity and reliability

#### Study population

According to the COSMIN guidelines, assessing structural validity requires a sample size of at least 6 times the number of items to obtain sufficient statistical power, and for an adequate assessment of the reliability, a sample size of 50–99 is advised [28]. The used CSD contains 9 items, and therefore, we aimed for 70 participants. Participants were recruited from the database of OIM Orthopedie, supplemented with participants of the feasibility study on 3D-printed hand orthoses [35]. The same in- and exclusion criteria were held as outlined earlier.

#### Procedure

After obtaining informed consent, the investigator collected demographical and clinical data of the participants. Subsequently, the D-CSD was sent digitally using Castor (Castor EDC, Amsterdam, the Netherlands) or by post (T1). The questionnaire was sent a second time two weeks after the first questionnaire was completed (T2). If necessary, a reminder was sent after one week.

#### Data analysis

Socio-demographics, clinical characteristics and mean (SD) D-CSD scores at T1 and T2 were summarized with descriptive statistics. Further, floor and ceiling effects were examined, which were defined as being present if at least 15% of participants reached the lowest or highest possible score, respectively [36].

##### Structural validity

Structural validity, an aspect of construct validity, is defined as ‘the degree to which the scores of a measurement instrument are an adequate reflection of the dimensionality of the construct to be measured’ [31]. The CSD was designed as a unidimensional construct. To determine the dimensionality of the D-CSD, a principal component analysis (PCA) was performed. To assess whether PCA was appropriate for the present data set, the Kaiser-Meyer-Olkin (KMO) value of sampling adequacy (threshold > 0.70), Bartlett’s value of Sphericity (threshold p < 0.05), and the determinant of correlation matrix were determined (threshold > 0.00001). The number of meaningful factors was determined with Horn parallel analysis (HPA) [37]. Thereafter, a confirmatory factor analysis (CFA) with Weighted Least Squares with Mean and Variance adjustment estimation was performed, to assess the fit of the factor model estimated by PCA. Sufficient evidence was considered for the determined dimensionality and thus a good model fit when the following criteria were met; (1) Comparative Fit Index (CFI) > 0.95, (2) Tucker-Lewis Index (TLI) > 0.95, (3) root mean square error of approximation (RMSEA) < 0.06, and (4) standardized root mean residuals (SRMR) < 0.08 [38].

##### Reliability

Reliability, ‘the degree to which the measurement is free from measurement error’, was determined by the measurement properties internal consistency, test-retest reliability, and measurement error [31]. For internal consistency (i.e. the degree of inter-relatedness among the items), Cronbach’s alpha was calculated. A Cronbach’s alpha ≥ 0.70 was considered to reflect good internal consistency [38]. To investigate test-retest reliability, the intra-class coefficient (ICC) and its 95% confidence interval (CI) were calculated using a two-way mixed effects model for a single measurement. Test-retest reliability was considered poor, moderate, good or excellent if the 95% CI of the ICC was less than 0.5, between 0.5 and 0.75, between 0.75 and 0.9, or greater than 0.90, respectively [39]. Systematic differences between test scores on the two occasions (đ) and the 95% CI were analyzed with paired-samples t-tests (for normally distributed outcomes) or Wilcoxon signed-rank tests (for non-normally distributed outcomes). To evaluate measurement error, a Bland-Altman plot was constructed and the 95% limits of agreement (LoA) were calculated (đ ± 1.96 × SD over the differences between test occasions) [40]. Also, the standard error of measurement (SEM) and smallest detectable change (SDC) were calculated. The SEM_agreement_, representing the limits for the smallest change that indicates a real change for a group of individuals, was calculated as √(variance occasions + variance error) [41, 42]. The SDC, indicating the amount of change at individual level that is real and not due to a potential measurement error, was determined using the formula: 1.96×SEM×√2 [42].

Statistical analyses were performed using R statistics’ version 4.0.3, packages psych, lavaan, BlandAltmanLeh and ggplot2 (R foundation for Statistical Computing, Vienna, Austria). P < 0.05 was considered significant. Missing values were not imputed. Since the response option ‘Don’t know/Not applicable’ was not scored numerically and therefore marked as missing, available case analysis was used in the factor analyses and Cronbach’s alpha calculation.

## Results

### Translation and content validity

#### Translation

The forward and backward translations showed minor variations in wording. Specifically, the expert committee questioned whether the Dutch translation of ‘durable’ in item 6 would be interpreted as sustainable. For item 2, another Dutch word for ‘manageable’ was chosen (in Dutch ‘te hanteren’). Translations of the response options were deemed clear. After reaching consensus, the developer of the CSD made two remarks on the pre-final version: (1) in the original CSD, negation was avoided to minimize response confusion. However, in the D-CSD, we choose to phrase a negative question for item 7, since the sentence would become too long and complex in Dutch, and (2) in line with the expert committee, the developer questioned the Dutch translation of ‘durable’ of item 6 since one backward translation was ‘sustainable’, which might be understood as being made of renewable materials. To prevent confusion, we added a synonym (wear-resistant) to this item in the final version of D-CSD.

#### Content validity

The mean (SD) age of the ten participants (9 females) was 58.7 (9.1) years (Table 2). Nine participants were native Dutch, and one participant was native English. The level of education ranged from lower vocational education to university.

All ten participants (100%) and the professionals indicated that the D-CSD items were relevant for the construct of interest, target population and context of use. Furthermore, the response options were considered appropriate.

Although two participants questioned one item (i.e. item 1 and item 7), ≥ 85% of the participants agreed that the instruction, items and response options were comprehensible. Also, the professionals agreed that the items were appropriately worded and that the response options matched the items.

Three out of ten participants (30%) missed an item about cleaning the orthosis. The professionals agreed on the relevance of this item since the orthosis is generally worn daily. Therefore, the item ‘My prosthesis/orthosis is easy to clean’ was formulated and positively judged on relevance and comprehensibility by three other participants. By adding this item to the final D-CSD, thus including 10 items (score range from 0 to 40 points, with a higher score indicating a higher satisfaction), the comprehensiveness was rated sufficient.

Since the relevance, comprehensibility, and comprehensiveness were all sufficient, the content validity of the D-CSD was judged sufficient.

### Structural validity and reliability

**Fig. 1:**
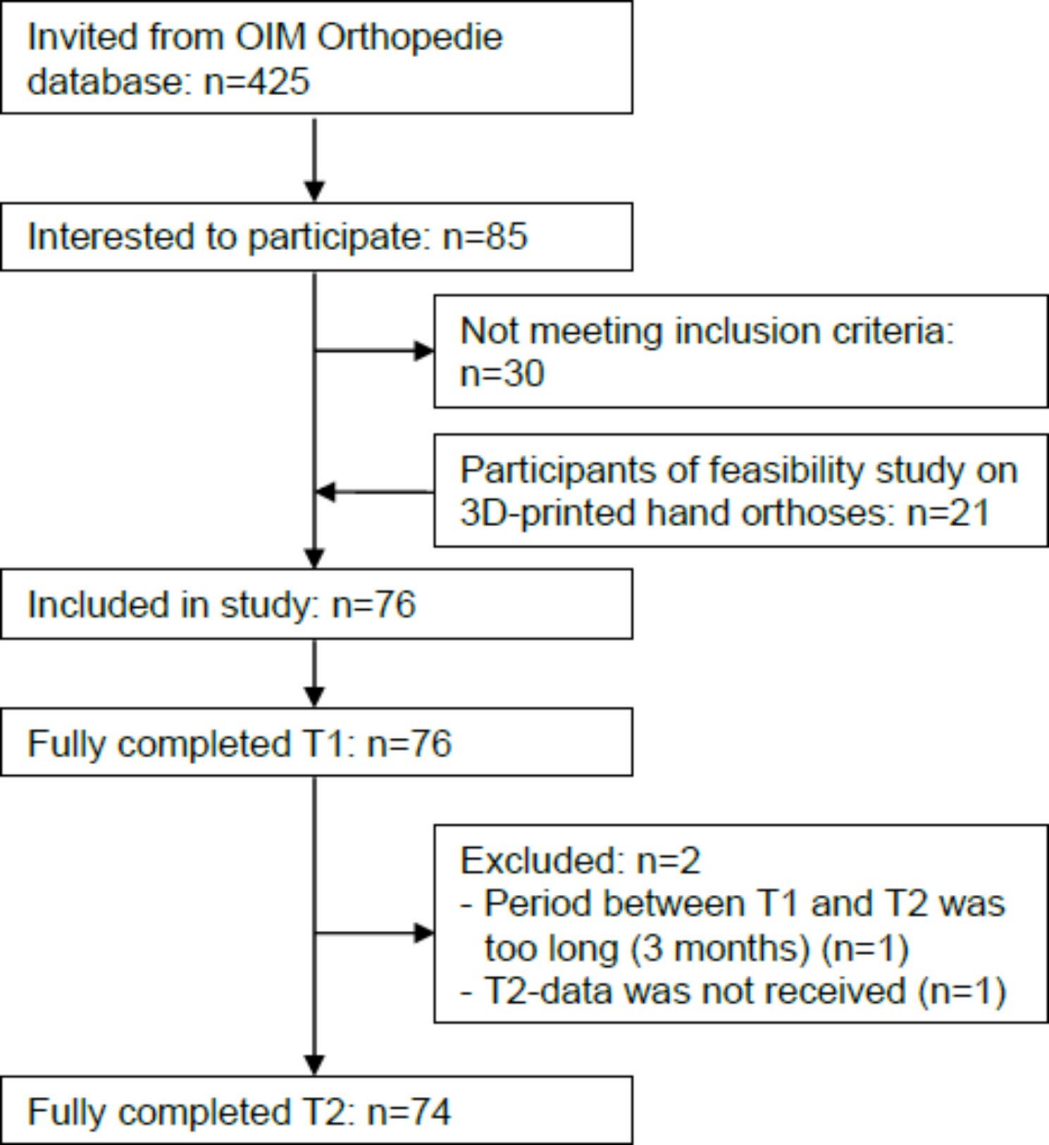
Participant flow chart

We invited 425 people from the OIM Orthopedie database, of whom 85 persons were interested to participate. Fifty-five persons met the in- and exclusion criteria. Combined with 21 participants of the feasibility study, 76 participants were included (Fig. 1). Their demographics and clinical characteristics are presented in Table 2. Because the period between T1 and T2 of one participant was 3 months and no T2 data was received from another participant, these two participants were excluded from the test-retest reliability analysis. Based on 74 participants, the mean (SD) D-CSD score at T1 and T2 was 26.8 points (6.47) and 25.9 points (6.37), respectively. No floor or ceiling effects were observed, since, respectively, none of the participants obtained the lowest possible score, and only 5% of the participants obtained the highest possible score on T1 and none on T2.

**Table 2:** Demographic and clinical characteristics of the participants

	Content validity (n = 10)	Structural validity and reliability (n = 76)
Age (years); mean (SD)	58.7 (9.1)	60.5 (10.8)
Gender; males/females	1 (10%) / 9 (90%)	15 (20%) / 61 (80%)
Reason chronic hand condition		
*injury*	-	2 (3%)
*musculoskeletal disorder*	7 (70%)	63 (83%)
*neuromuscular disorder*	3 (30%)	3 (4%)
*neurological disorder*	-	8 (11%)
Orthosis; unilateral/bilateral	7 (70%) / 3 (30%)	36 (47%) / 40 (53%)
Type of orthosis*		
*thumb orthosis*	7 (54%)	40 (34%)
*wrist orthosis*	1 (8%)	35 (30%)
*wrist-thumb orthosis*	5 (38%)	41 (35%)
Wearing days per week*		
*6–7 days*	6 (46%)	68 (59%)
*4–5 days*	2 (15%)	21 (18%)
*2–3 days*	4 (31%)	21 (18%)
*1 day*	1 (8%)	6 (5%)
Wearing time per day*		
*24 h*	1 (8%)	4 (3%)
*During daytime*	3 (23%)	47 (41%)
*During strenuous activities*	8 (62%)	56 (48%)
*Other (e.g. pain)*	1 (8%)	9 (8%)

NOTE. Values are n (%), unless otherwise indicated. SD = standard deviation

* Based on the total number of orthoses worn by the participants

#### Structural validity

The KMO indicated good sampling adequacy (KMO = 0.82), Bartlett’s test was significant (p < 0.001), and the correlation matrix determinant was 0.069. HPA indicated the presence of one factor. PCA showed item loadings ranging from 0.44 to 0.72 (Table 3). The factor explained 39% of the variance. CFA demonstrated a good one-factor model fit, with all fit indices above the reference criteria (CFI = 1.00, TLI = 1.03, RMSEA < 0.001, SRMR = 0.06).

**Table 3:** Factor loadings of D-CSD items

D-CSD item	Factor loadings
1. My prosthesis/orthosis fits well	0.65
2. The weight of my prosthesis/orthosis is manageable	0.63
3. My prosthesis/orthosis is comfortable throughout the day	0.65
4. It is easy to put on my prosthesis/orthosis	0.67
5. My prosthesis/orthosis looks good	0.69
6. My prosthesis/orthosis is durable (wear-resistant)	0.63
7. My clothes are free of wear and tear	0.51
8. My skin is free of abrasion and irritation	0.72
9. My prosthesis/orthosis is pain free to wear	0.58
10. My prosthesis/orthosis is easy to clean	0.44

#### Reliability

**Fig. 2:**
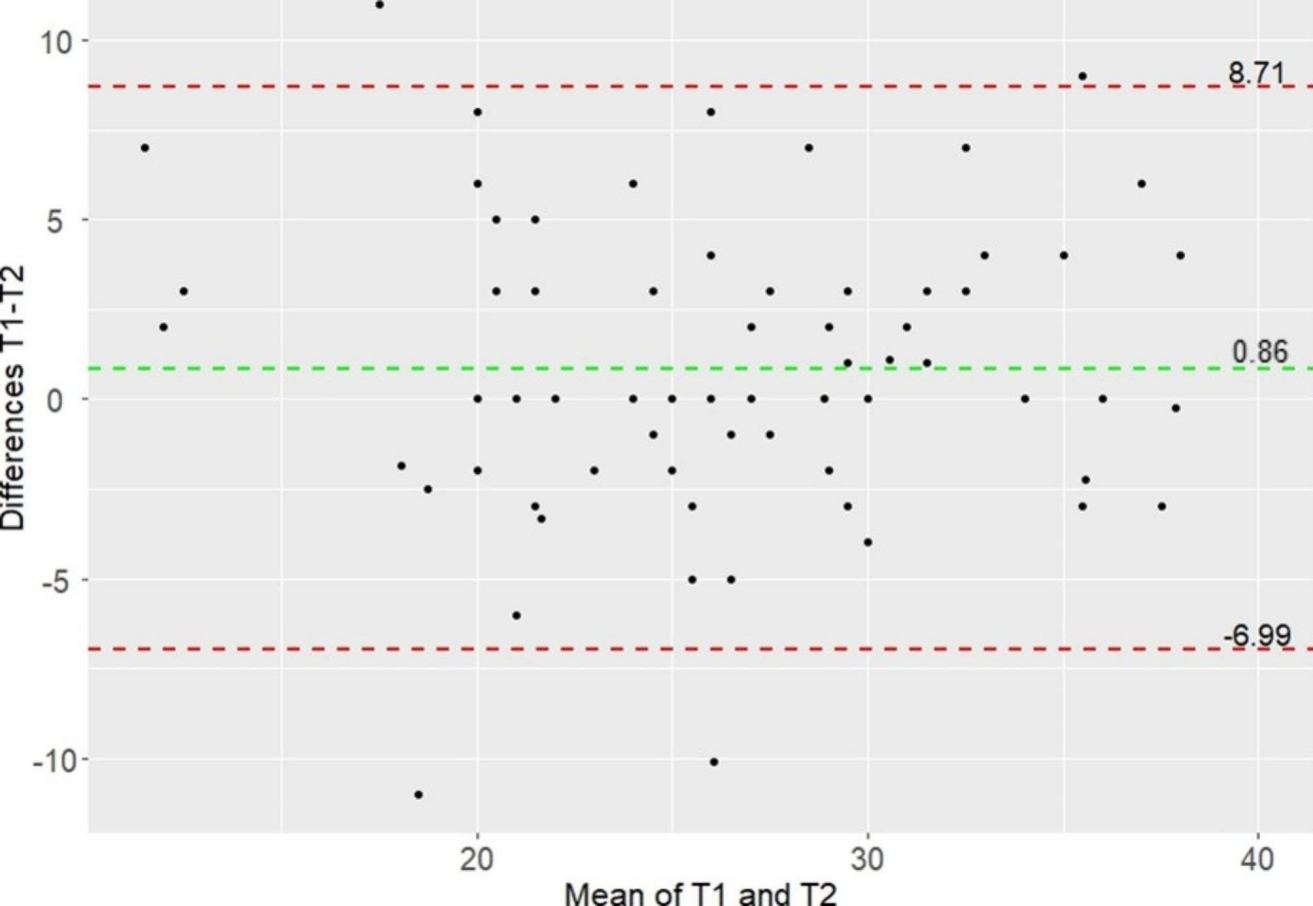
Bland-Altman plot. The green line indicates the mean difference between T1 and T2. Red lines indicate the 95% limits of agreement

Cronbach’s alpha was 0.82 (95%CI 0.75–0.87), indicating good internal consistency. Dropping an item did not improve the internal consistency (range with one item deleted: 0.78–0.82). There was no significant difference between the two occasions (mean (SD) difference: 0.86 points (4.00); 95%CI -0.06-1.79; p = 0.07). The ICC was 0.81 (95%CI 0.71–0.87), indicating moderate to good test-retest reliability. The Bland-Altman plot, including the 95% LoA (-6.99 to 8.71), is shown in Fig. 2. The SEM and SDC were 2.88 and 7.98 points, respectively.

## DISCUSSION

The results of this study showed sufficient content and structural validity of the D-CSD in our sample of chronic users of hand orthoses. Further, good internal consistency, moderate to good test-retest reliability and acceptable measurement error were found. Sensitivity to detect changes on individual level was limited.

With regard to content validity, the components relevance, comprehensibility and comprehensiveness were all rated sufficient. Although one item was knowingly negatively phrased, comprehensibility was not adversely affected. Some participants indicated missing an item on cleaning of the orthosis, which was therefore included in the final version of the D-CSD. Including this item did not affect the internal consistency, since the Cronbach’s alpha remained constant when this item was dropped. Although PCA showed that this item had the lowest factor loading (0.44), it was above the cut-off value of 0.40, and thus considered acceptably correlated to the construct measured [43]. A recent scoping review on the psychometric properties of the CSD suggested to discard item 7 on ‘wear and tear on clothing’ [44], as it did not fit the model in Rasch analysis in three studies in lower and upper limb orthotic and prosthetic users [13, 17, 26]. Based on our clinical experience, clothing wear and tear is a well-known problem in the target population, which was also indicated by participants during the cognitive debriefing. Furthermore, our PCA showed adequate factor loading of this item, and dropping it would have lowered the internal consistency, supporting our decision to retain this item in the D-CSD.

Regarding structural validity, and in line with our hypothesis, the results of the PCA indicated a one-factor model, consistent with the English 9-item CSD, and which has also been demonstrated in five other studies in persons with spine, and upper and lower extremity orthotics and prosthetics [14, 18, 21–23, 26]. The explained variance of 39% is within the range of 37–88% reported in these studies. We furthermore confirmed the unidimensionality of the D-CSD as the CFA resulted in adequate one-factor model fit indices. No other studies have been conducted using CFA in assessing the structural validity of the CSD to compare our results with.

The D-CSD showed good internal consistency, which is in line with our hypothesis and comparable to earlier findings in persons with spine, and upper and lower extremity orthotics and prosthetics [14, 18, 19, 21–23]. Furthermore, the D-CSD showed moderate to good test-retest reliability, indicating that the D-CSD can adequately distinguish persons with high and low orthosis satisfaction scores. The ICC found in our study is comparable with studies on the Persian, Swedish and Turkish CSD [19, 20, 23], and much higher than the reported ICC of 0.50 for the English CSD [24]. Reliability in this latter study however, was assessed in a sample of veterans wearing unilateral lower limb prostheses, who, compared to our sample of chronic hand orthotic users with a variety of diagnoses and impairments, might represent a more homogeneous group. Probably, this resulted in less between-subject variance, thereby lowering the ICC. Overall, it should be noted that previous studies examining the psychometric properties of the CSD, including reliability, used different populations, sample sizes, and CSD-versions (i.e. number of items, response options and scoring systems), which limits a fair comparison with our results.

Despite good test-retest reliability and an acceptable SEM of 2.88 points (11% of the pooled mean D-CSD score), the SDC was relatively high, i.e. an individual needs to change > 7.98 points (30% of the pooled mean D-CSD score) to ensure the detection of a true change. Although different populations, sum scores and SEM calculations were used, this is within the range of 16–34% of the mean CSD score reported in earlier studies [19, 20, 23, 24]. Ideally, the SDC should not exceed the minimal important change (MIC), a threshold for a minimal within-person change over time above which persons perceive themselves importantly changed [38]. Unfortunately, no research has been performed on the MIC of the CSD. As a rule of thumb, it has been suggested that the MIC can be estimated as 10% of the maximum score of a measurement [45]. In our study, this would result in a MIC of 4 points, which is far below our SDC of 7.98 points, indicating limited applicability of the CSD to detect importantly changes in orthosis satisfaction on individual level. For clinical practice, in order to detect smaller changes or changes below the MIC, the outcome measure requires a smaller SDC. This can be achieved by increasing the number of measurements to overcome the problem of large measurement error [42]. Future research should focus on assessing the effect of using multiple repeated measurements over time on the SDC in so-called G-studies and D-studies [46], and on determining the MIC of the CSD in chronic hand orthotic users to compare these two outcomes adequately. Furthermore, as we investigated the validity and reliability of the D-CSD in hand orthotic users, yet the CSD also targets lower extremity orthotic users and upper and lower extremity prosthetic users. Future studies are needed to investigate the psychometric properties of the D-CSD in these populations.

### Strengths and limitations

A strength of our study was the specific attention given to the content validity. This type of validity is considered the most important measurement property, indicating whether questionnaire items are relevant, comprehensive, and comprehensible with respect to the construct of interest and study population [32], which was shown in our study. Furthermore, since this study was conducted in a heterogeneous sample (i.e. diversity of diagnoses) of chronic hand orthotic users, wearing the three most commonly prescribed types of hand orthoses, we are confident that the results can be generalized to the population of chronic hand orthotic users at large.

Our study also has some limitations. Although we invited 425 persons to participate in our study, no more than 85 persons were interested to participate. Due to this low response rate (20%), combined with the 21 participants specifically willing to participate in our feasibility study on 3D-printed orthoses, selection bias could have occurred. Besides, a higher sample size, ideally ≥ 100 participants [28], could have resulted in higher precision of the validity and reliability estimates.

## CONCLUSION

We showed sufficient content validity and structural validity, and good reliability of the D-CSD in Dutch chronic hand orthotic users. Given the relatively high SDC, sensitivity to detect changes in orthosis satisfaction over time on individual level is limited. Yet, based on the SEM, the D-CSD is considered a useful tool to assess satisfaction of hand orthoses on group level in this population.

## Data Availability

The datasets used and/or analysed in the current study are available from the corresponding author on reasonable request.

## Abbreviations

CFA: confirmatory factor analysis
CFI: Comparative Fit Index
CI: Confidence Interval
COSMIN: COnsensus-based Standards for the selection of health Measurement Instruments
(D)-CSD: (Dutch) Client Satisfaction with Device
(D)-QUEST: (Dutch) Quebec User Evaluation of Satisfaction with assistive Technology
HPA: Horn parallel analysis
ICC: intra-class coefficient
KMO: Kaiser–Meyer–Olkin
LoA: limits of agreement
OPUS: Orthotics and Prosthetics Users’ Survey
PCA: principal component analysis
PROM: patient-reported outcome measure
RMSEA: Root Mean Square Error of Approximation
SD: standard deviation
SDC: smallest detectable change
SEM: standard error of measurement
SRMR: Standardized Root Mean Residual
TLI: Tucker Lewis Index
